# The impact of hypertension follow-up management on the choices of signing up family doctor contract services: does socioeconomic status matter?

**DOI:** 10.1186/s12875-024-02383-8

**Published:** 2024-04-24

**Authors:** Xuehong Wang, Jie Chu, Dan Zhao, Tingting Gao, Jingjing Luo, Xueqing Wang, Shujun Chai, Jiayan Li, Jingjie Sun, Peilong Li, Chengchao Zhou

**Affiliations:** 1 Centre for Health Management and Policy Research, School of Public Health Cheeloo College of Medicine, Shandong University, Jinan, 250012 China; 2 Institute of Health and Elderly Care, Shandong University, Jinan, 250012 China; 3 Shandong Center for Disease Control and Prevention, Jinan, 250012 China; 4 Shandong Health Commission Medical Management Service Center, Jinan, 250012 China; 5 NHC Key Lab of Health Economics and Policy Research, Shandong University, Jinan, 250012 China

**Keywords:** Hypertension follow-up management, Family doctor contract services, Socioeconomic status

## Abstract

**Background:**

This study aimed to explore the association between hypertension follow-up management and family doctor contract services, as well as to examine whether socioeconomic status (SES) had an interaction effect on this relationship among older adults in China.

**Methods:**

We used data from the sixth National Health Service Survey of Shandong Province, China, including 3,112 older adults (age ≥ 60 years) with hypertension in 2018. Logistic regression models and a margins plot were used to analyze the role of SES in the relationship between hypertension follow-up management and family doctor contract services.

**Results:**

The regular hypertension follow-up management rate and family doctor contracting rate were 81.8% and 70.9%, respectively, among older adults with hypertension. We found that participants with regular hypertension follow-up management were more likely to sign family doctor contract services (OR=1.28, 95%CI: 1.04, 1.58, *P*=0.018). The interaction effect occurred in the groups who lived in rural areas (OR=1.55, 95%CI: 1.02, 2.35), with high education level (OR=0.53, 95%CI: 0.32, 0.88) and had high incomes (OR=0.53, 95%CI: 0.35, 0.81).

**Conclusions:**

Our findings suggested that regular hypertension follow-up management was associated with family doctor contract services and SES influenced this relationship. Primary health care should improve the contracting rate of family doctors by strengthening follow-up management of chronic diseases. Family doctors should focus on improving services quality and enriching the content of service packages especially for older adults with higher income and education level.

## Introduction

Family doctor contract services refer to the model of proactive, continuous and comprehensive health accountability management provided by family doctors signing service contracts with residents [1], which has become the basis of the hierarchical medical system in China [2]. In addition to providing basic medical and public health services to contracted residents, family doctors also provide long-term follow-up and case-based management for patients with chronic diseases such as hypertension and diabetes [3]. The role of family doctors in improving the health status of the residents is gradually being proven [4, 5]. As the key population of family doctor contract services, older adults with chronic conditions have a higher need for health management [6], and they are more likely to contract with family doctors [7, 8]. It is now well established from a variety of studies, that a higher contracting rate is associated with better chronic disease self-management and lower health care costs [9–11]. However, the current implementation of family doctor services in China is at its early stage, the contract coverage is rather low and the quality of services needs to be improved [12–14]. It is important to increase the contracting rate of family doctors for older adults, especially for patients with chronic disease, to actively respond to the aging trend and realize the Healthy China initiative.

Hypertension is one of the most common chronic diseases leading to more serious illnesses, and is the largest single risk factor for all-cause mortality [15]. There is a steady increase in the prevalence of hypertension in China with a prevalence of over 45% among older adults by 2015 [16]. Studies have shown that hypertension can be prevented and improved through effective management [17]. Follow-up visits of hypertension are regular clinic activities for hypertensive patients to achieve blood pressure control, emphasized by a series of hypertension guidelines [18]. Regular follow-up visits of hypertension could enhance blood pressure monitoring and improve medication adherence, thereby preventing complications and reducing medical expenses [19]. Chinese national standards suggest 4 times per year for hypertension follow-up care delivery as an important part of the national basic public health services [20]. Actually, patients with hypertension may be diagnosed and initially followed up in large hospitals and clinics, considered to be "passive" health management for disease causes [21]. Further, a high frequency of follow-up services improved the health level and health awareness of hypertensive patients leading residents proactively to seek health management and sign with family doctors [22, 23]. Primary health workers have also contributed greatly to increasing family doctor contracting rates for patients with hypertension follow-up management [13, 24]. In addition, the systematic health management through the family doctor contract services actually enrich the content of primary health care than only hypertension follow-up management [6]. These findings indicate that there may be a relationship between regular hypertension follow-up management and family doctor contract services. This study will further verify the relationship among older adults in China to facilitate the development of the family doctor services system.

Socioeconomic status (SES), combined by several variables including education, occupation, family residence and income, is one of the most important factors influencing health decisions [25]. There is a considerable difference in economic development level, occupational structure and health care system construction between urban and rural areas, living residence is a representative indication of SES in China [26]. In addition, older adults are predominantly retired and jobless, previous studies have chosen family residence, education and income to measure older adults’ socioeconomic status [27, 28]. In the Anderson’ s health behavior model, SES belongs to personal characteristics and is the underlying component that influence health service activities [29]. It has been found that residents' choices of family doctor contracting may vary by SES [10]. According to a survey based on 31 provinces and cities in China, the lower level of education, higher level of income and living in rural area were contributing factors for contracting with family doctors [30].The impact of SES on the follow-up management of hypertension has also been demonstrated. Another study has proved that older adults living in rural areas were more likely to adopt hypertension follow-up services, and residents with high-level education were less likely to use the services [31]. However, it is still not clear whether the association between regular hypertension follow-up management and family doctor contract services among older adults varies across different SES.

The aims of this study are as follows: 1) to explore the relationship between regular hypertension follow-up management and family doctor contract services among older adults; 2) to examine whether SES had an interaction effect on the relationship. We hypothesize that older adults with regular hypertension follow-up management prefer to sign family doctor contract services, and this relationship varied by SES.

## Methods

### Data and sample

Data for this study were drawn from the Health Service Survey of Shandong Province in 2018, which was part of the National Health Service Survey (NHSS). As a nationally representative survey, NHSS has been conducted every five years since 1993 to understand the basic health status of the population and the situation of health care utilization [32]. More details about the interview have been published in previous studies [33–35]. The survey used a multistage cluster sampling method to select 100 townships of which 20 counties were randomly chosen within 137 counties of Shandong Province. Then two sample villages (communities) were randomly selected from each township and each sample village had 60 or more households involved in the survey. Finally, 12,938 households and 35,264 individuals participated in this research.

Data were collected using face-to-face interviews and structured questionnaires were adopted to interview all the participants. We restricted the age of respondents to 60 years and older as the study sample. Finally, a total of 8,642 older adults were included in the analysis after excluding participants with dementia. Further screening out those older adults diagnosed with hypertension and excluding samples with missing values on main variables, our final analysis sample included 3,112 participants.

### Measure

#### Family doctor contract services

Family doctor contract services status was a binary variable measured by the question: “Did you contract family doctor services?” Respondents answer “Yes” (coded as 1) and “No” (coded as 0).

#### Hypertension follow-up management

Participants with diagnosed hypertension should answer the question about hypertension follow-up management: “How many times did you receive hypertension follow-up services in the last 12 months?”. The answers are the following six types: “Once”, “Twice”, “Three times”, “Four times”, “Five times and more” or “Not followed up”. According to the National Basic Public Health Service (3^rd^ edition) specification, patients with primary hypertension should be provided with at least four times face-to-face follow-up services per year. We defined regular hypertension follow-up as receiving more than four times of hypertension follow-up services per year (coded as 1). Irregular hypertension follow-up was defined as receiving less than four times follow-up visits per year (coded as 0).

#### Socioeconomic status

The SES was measured by family residence, education and annual household income in this study. The family residence was divided into urban and rural areas. The education attainment was recorded as illiteracy, primary school or below, and secondary school or above. Household income was self-reported and classified into two groups: low household income (below the average) and high household income (above the average).

#### Control variables

We identified other correlates on the basis of previous research. The controlled variables consisted of gender (male /female), age (years), marital status [single (unmarried, divorced, widowed and others) /married], need caregiving (yes /no), smoking status (yes /no), drinking status (yes /no), physical examination (yes /no), living arrangements (alone /with others), blood pressure situation (abnormal /normal), medication for hypertension (irregular /regular).

### Statistical analysis

All statistical analyses were performed with Stata 14.0 (Stata Corp, College Station, TX, USA). We used frequency and percentage to describe the characteristics of the participants. Student’s t-tests and chi-square tests were used to compare the family doctor contract services status across different groups. Then we used logistic regression models to examine the association between regular hypertension follow-up management and family doctor contract services. Model 1 of the regression analysis included independent variables and all confounding variables. Further, we included the interaction terms in Model 2, Model 3 and Model 4 to test the effects of SES (living region, educational attainment and household income, separately). Furthermore, the margins plot was employed to illustrate the prediction of family doctor contract status by hypertension management and SES. The confidence interval reported in this study was calculated at the 95% level. *P-*values less than 0.05 (two-tailed) were considered statistically significant.

## Results

### Characteristics of participants

Table 1 shows the basic characteristics of the participants. A total of 3,112 older adults with hypertension were enrolled in this study. Of them, 1,738 (55.8%) were female and 2,206 (70.9%) had signed the family doctor contract services. The average age of the participants was 69.4 (±6.7) years old and most of them were married. Compared with older adults who did not sign the family doctor contract, participants who signed the services had high proportion of urban residency, low income, smoking and normal blood pressure situation, and they were older, more educated and had physical examinations.

**Table 1:** Descriptive statistics of signed status of family doctor contract services among older adults with hypertension in Shandong, China (*N*=3,112)

**Variable**	**N (%)**	**Contract status**		**χ**^**2**^**/ t-test**	***P*-Value**
		**No (%)**	**Yes (%)**		
*N*=3,112		906 (29.1)	2,206 (70.9)		
**Age (Mean±SD)**	69.4 ±6.7	69.3 ±7.1	69.5 ±6.5	11.2	0.001
**Gender**				1.8	0.176
Male	1,374 (44.2)	383 (42.3)	991 (44.9)		
Female	1,738 (55.8)	523 (57.7)	1,215 (55.1)		
**Marital status**				0.8	0.363
Single^a^	557 (17.9)	171 (18.9)	386 (17.5)		
Married	2,555 (82.1)	735 (81.1)	1,820 (82.5)		
**Region**				13.5	<0.001
Urban	1,544 (49.6)	403 (44.5)	1,141 (51.7)		
Rural	1,568 (50.4)	503 (55.5)	1,065 (48.3)		
**Education**				11.9	0.003
Illiteracy	982 (31.6)	249 (27.5)	733 (33.2)		
Primary or below	1,031 (33.1)	303 (33.4)	728 (33.0)		
Secondary school or above	1,099 (35.3)	354 (39.1)	745 (33.8)		
**Annual household income**				3.9	0.047
Low	1,969 (63.3)	549 (60.6)	1,420 (64.4)		
High	1,143 (36.7)	357 (39.4)	786 (35.6)		
**Need caregiving**				1.4	0.232
No	2,706 (86.9)	798 (88.1)	2,206 (86.5)		
Yes	406 (13.1)	108 (11.9)	298 (13.5)		
**Smoking status**				4.5	0.033
No	2,649 (85.1)	752 (83.0)	1,897 (86.0)		
Yes	463 (14.9)	154 (17.0)	309 (14.0)		
**Drinking status**				3.4	0.064
No	2,453 (78.8)	695 (76.7)	1,758 (79.7)		
Yes	659 (21.1)	211 (23.3)	448 (20.3)		
**Physical examination**				135.9	<0.001
No	694 (22.3)	325 (35.9)	369 (16.7)		
Yes	2,418 (77.7)	581 (64.1)	1,837 (83.3)		
**Living arrangements**				0.1	0.764
Alone	366 (11.8)	109 (12.0)	257 (11.6)		
With others	2,746 (88.2)	797 (88.0)	1,949 (88.4)		
**Blood pressure situation**^**b**^				10.0	0.002
Abnormal	1,324 (42.5)	425 (46.9)	899 (40.8)		
Normal	1,788 (57.5)	481 (53.1)	1307 (59.2)		
**Medication for hypertension**^**c**^				<0.1	0.958
Irregular	764 (24.5)	223 (24.6)	541 (24.5)		
Regular	2,348 (75.5)	683 (75.4)	1,665 (75.5)		
**Hypertension follow-up management**^**d**^				2.7	0.103
Irregular	567 (18.2)	181 (20.0)	386 (17.5)		
Regular	2,545 (81.8)	725 (80.0)	1,820 (82.5)		

^a^Singles include those who are unmarried (27, 0.87%), divorced (8, 0.26%), widowed (520, 16.71%) and others (2, 0.06%)

^b^Blood pressure situation refers to the most recent blood pressure at the time of the survey

^c^Regular refers to the patient regularly taken hypertension medication following the medical order

^d^Whether it meets the national follow-up standards for hypertension (4 times a year)

### Association between regular hypertension follow-up management and family doctor contract services

Table 2 shows the influence of hypertension follow-up management on family doctor contract services status among older adults. In Model 1, logistic regression confirmed that there was a positive association between hypertension follow-up management and family doctor contract status. After adjusting for all control variables, older adults with regular hypertension follow-up management (OR=1.28, 95%CI: 1.04, 1.58) were more likely to contract family doctor services.

**Table 2:** Association between regular hypertension follow-up management and family doctor contract services status among older adults with hypertension in Shandong, China, 2018

**Characteristics**	**Model 1**^**a**^		**Model 2**^**b**^		**Model 3**^**c**^		**Model 4**^**d**^	
	**OR (95%CI)**	***P*-value**	**OR (95%CI)**	***P*-value**	**OR (95%CI)**	***P*-value**	**OR (95%CI)**	***P*-value**
*Main terms*								
Follow-up management (Irregular ^Ref^)								
Regular	1.28 (1.04, 1.58)	0.018	1.07 (0.81, 1.41)	0.642	1.77 (1.21, 2.61)	0.004	1.65 (1.27, 2.15)	<0.001
Education (Illiteracy ^Ref^)								
Primary or below	0.74 (0.60, 0.91)	0.005	0.74 (0.59, 0.91)	0.005	0.89 (0.56, 1.43)	0.637	0.73 (0.59, 0.91)	0.005
Secondary school or above	0.58 (0.46, 0.74)	<0.001	0.58 (0.45, 0.74)	<0.001	0.97 (0.60, 1.57)	0.907	0.58 (0.45, 0.74)	<0.001
Region (Urban ^Ref^)								
Rural	0.62 (0.52. 0.74)	<0.001	0.43 (0.29, 0.64)	<0.001	0.62 (0.52, 0.74)	<0.001	0.61 (0.51, 0.73)	<0.001
Annual household income (Low ^Ref^)								
High	0.81 (0.67, 0.98)	0.027	0.81 (0.67, 0.98)	0.030	0.81 (0.67, 0.97)	0.024	1.35 (0.91, 1.99)	0.131
*Interaction term*								
Follow-up management×Region								
Regular×Rural			1.55 (1.02, 2.35)	0.041				
Follow-up management×Education								
Regular×Primary or below					0.80 (0.47, 1.34)	0.391		
Regular×Secondary school or above					0.53 (0.32, 0.88)	0.015		
Follow-up management×Income								
Regular×High							0.53 (0.35, 0.81)	0.003
*Control variables*								
Gender (Male ^Ref^)								
Female	0.62 (0.50, 0.77)	<0.001	0.62 (0.50, 0.76)	<0.001	0.62 (0.50, 0.76)	<0.001	0.61 (0.49, 0.76)	<0.001
Age			0.99 (0.97, 1.00)	0.032	0.99 (0.97, 1.00)	0.033	0.99 (0.97, 1.00)	0.033
Marital status^e^ (Single ^Ref^)								
Married	1.09 (0.83, 1.44)	0.538	1.08 (0.82, 1.43)	0.569	1.07 (0.81, 1.42)	0.622	1.07 (0.81, 1.41)	0.627
Need caregiving (No ^Ref^)								
Yes	0.83 (0.64, 1.06)	0.137	0.82 (0.64, 1.06)	0.125	0.82 (0.64, 1.06)	0.130	0.83 (0.64, 1.06)	0.142
Cigarette smoking (No ^Ref^)								
Yes	0.81 (0.63, 1.04)	0.096	0.81 (0.63, 1.04)	0.103	0.81 (0.63, 1.05)	0.114	0.81 (0.63, 1.04)	0.100
Alcohol drinking (No ^Ref^)								
Yes	0.78 (0.62, 0.99)	0.040	0.78 (0.62, 1.00)	0.041	0.78 (0.61, 0.98)	0.036	0.78 (0.61, 0.98)	0.035
Health screening (No ^Ref^)								
Yes	2.92 (2.43, 3.50)	<0.001	2.90 (2.41, 3.48)	<0.001	2.91 (2.43, 3.50)	<0.001	2.89 (2.41, 3.48)	<0.001
Living arrangements (With others ^Ref^)								
Alone	0.99 (0.72, 1.36)	0.966	0.99 (0.72, 1.36)	0.958	0.98 (0.72, 1.35)	0.922	0.98 (0.72, 1.35)	0.918
Blood pressure situation (Abnormal ^Ref^)								
Normal	1.36 (1.16, 1.60)	<0.001	1.36 (1.15, 1.60)	<0.001	1.36 (1.16, 1.61)	<0.001	1.35 (1.15, 1.60)	<0.001
Medication (Irregular ^Ref^)								
Regular	0.98 (0.81, 1.19)	0.858	0.98 (0.81, 1.19)	0.860	0.98 (0.81, 1.19)	0.860	0.98 (0.81, 1.19)	0.844

^a^Adjusted for all control variables

^b^Adjusted for model 2 criteria and the interaction between hypertension follow-up management and region

^c^Adjusted for model 3 criteria and the interaction between hypertension follow-up management and education

^d^Adjusted for model 4 criteria and the interaction between hypertension follow-up management and household income

^e^Singles include those who are unmarried (27, 0.87%), divorced (8, 0.26%), widowed (520, 16.71%) and others (2, 0.06%)

### The interaction effect between regular hypertension follow-up management and socioeconomic status on family doctor contract services

**Fig. 1:**
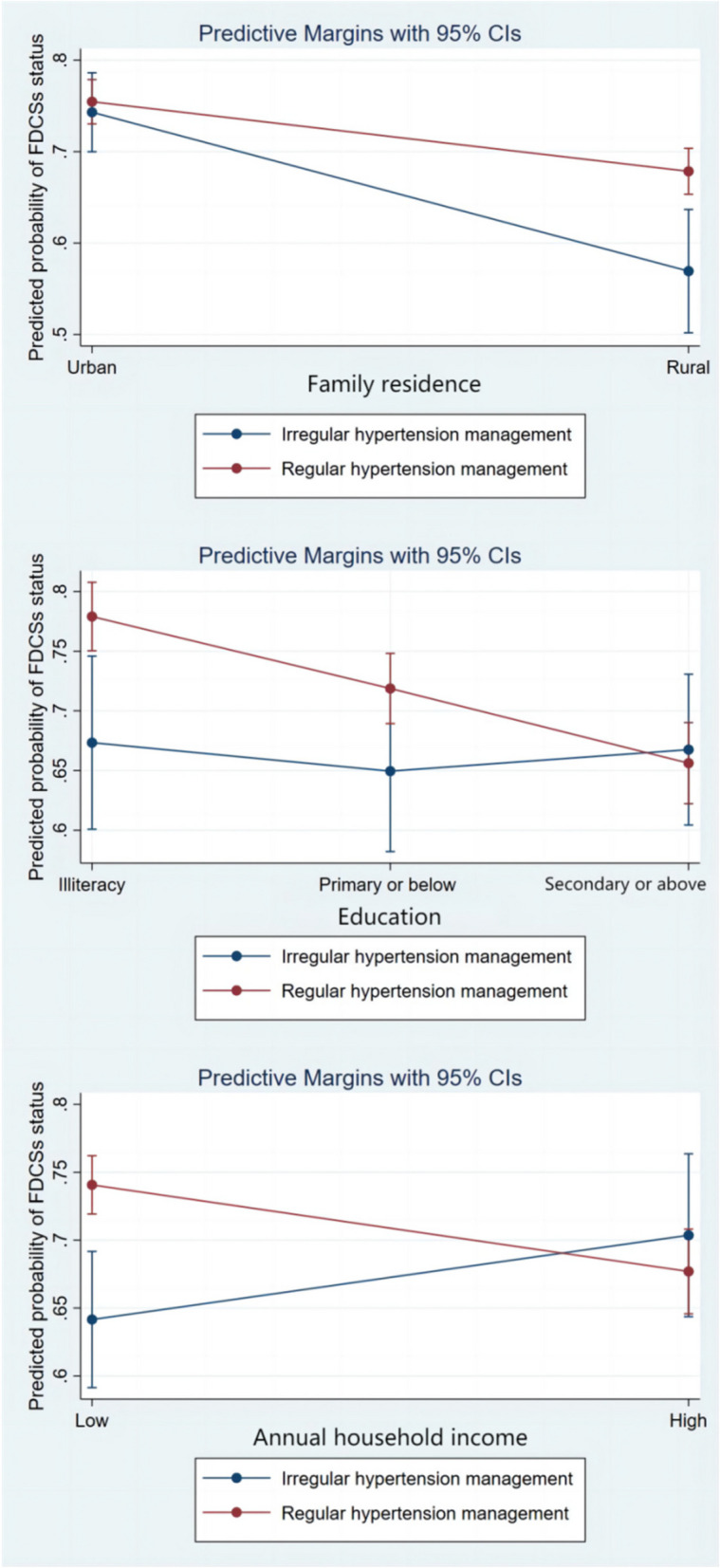
Interaction between hypertension management and socioeconomic status in the prediction of family doctor contract services sign status

Table 2 also includes the interaction term of regular hypertension follow-up management and SES to explore whether the relationship between regular hypertension follow-up management and family doctor contract services status varied by SES. Model 2 shows that older adults with regular hypertension follow-up management and rural living areas were more willing to sign the family doctor services (OR=1.55, 95%CI: 1.02, 2.35) than urban residents with irregular hypertension follow-up management. Model 3 shows that compared with illiterate older adults with irregular hypertension follow-up management, participants with high education (secondary school or above) and regular hypertension follow-up management had lower family doctor services contracting rates (OR=0.53, 95%CI: 0.32, 0.88). In Model 4, older adults in the high-income group with regular hypertension follow-up management were less likely to sign up for the services (OR=0.53, 95%CI: 0.35, 0.81) than those in the low-income group and with irregular hypertension follow-up management. In addition, Fig. 1 visualizes the marginal effect of regular hypertension follow-up management in different SES.

## Discussion

To the best of our knowledge, this is the first study to explore the role of SES in the relationship between hypertension follow-up management and family doctor contract services among older adults. Our results revealed that older adults with regular hypertension follow-up management were more willing to sign for family doctor contract services, as well as the interaction effect of SES in this relationship.

Our study found that regular hypertension follow-up management was positively associated with contracting family doctor services among older adults. Older adults with regular hypertension follow-up management preferred to sign family doctor contract services, which was similar to the findings of previous studies on primary health services [36, 37]. This result can be explained in terms of both supply and demand characteristics of health services. First, primary healthcare providers who offered hypertension follow-up management services were also important members of the family doctor team, and they played a critical role in promoting family doctor contracting [22]. The contracting rate and the percentage of key populations were important indicators for the performance assessment of family doctors [38, 39], which became a probable reason for primary health providers to introduce and promote family doctor contract services to older adults with hypertension. Residents who have received publicity for the family doctor contract policy showed a higher willingness to contract with family doctors, especially older adults with chronic diseases such as hypertension [13, 40]. Second, from the aspect of health service demand, hypertension, as one of the most common chronic diseases, is often accompanied by complications such as coronary heart disease, requiring more medical care and health management [17, 41]. Regular hypertension follow-up management sparks more health management needs among older adults. Family doctor contract services are effective in managing chronic diseases and can reduce healthcare expenditures [42]. Moreover, older adults who contracted family doctors can receive home visit services, making blood pressure management more convenient.

We also found that the association between hypertension follow-up management and family doctor contract services varied by socioeconomic status. It has been demonstrated that different SES affect the utilization of health services in previous studies. In this study, living in rural areas promoted older adults with regular hypertension management to contract family doctor services, while participants with high-levels of education and income were less likely to contract the services. Compared with urban areas, rural areas had scarcer medical resources [26, 43] , and hypertension follow-up services were also basically provided by village doctors who serve as family doctors [44]. Family doctor services can exactly provide the basic medical services and the health management services for rural older adults with chronic diseases [4, 45]. Traditional interpersonal communication style and the mutual trust made family doctors’ home visits more acceptable in rural areas [46]. In addition, the home visits provided by family doctors could improve the spatial accessibility of healthcare in rural areas, making older adults more willing to sign family doctor contract services.

Interestingly, older adults with a higher education level of secondary school or above were unwilling to sign up with family doctors after receiving regular hypertension follow-up services, which is contrary to previous studies [40, 47]. Since well-educated residents with a higher level of health literacy, should better grasp the policy of family doctor contract services. However, regular follow-up hypertension management helped well-educated older adults pay more attention to their health management, with distrust of primary care institutions, they preferred to go to high-level hospitals for further treatment [48]. As for the impact of income, we found that high-income older adults with regular hypertension follow-up management were less likely to sign up family doctors. Prior research also indicated that high-income groups used fewer primary health care services [49]. For high-income residents, regular hypertension management allows them to better control their blood pressure and seek better treatment measures. The basic family doctor contract services package was simple and couldn’t meet the personalized and private health management demand of high-income people. Additionally, previous qualitative studies have found that residents' distrust of family doctors’ competence is an important factor influencing contracting rate [50, 51], and such concerns may be more pronounced among highly educated and high-income populations.

Our findings provided some evidence for promoting older hypertension patients’ health management. First, effective follow-up services should be provided to promote the health status of older adults with chronic diseases. Second, family doctors should pay more attention to higher quality of health care services for older adults with hypertension through long-term prescriptions and regular follow-up services. Additionally, the service packages of family doctor contract should be well-designed to meet different health needs including some expectations claimed by high-educated and high-income groups.

There are a few limitations in this study. On the one hand, as a cross-sectional study, it could only explain the association, not the causality, between hypertension follow-up management and family doctor contract status. Longitudinal designs can be used in the future to confirm this relationship. On the other hand, data such as the number of hypertension follow-up management visits were self-reported by participants and may be subject to recall bias. Electronic records can be used in the future instead of self-reported.

## Conclusions

This study demonstrated that older adults with regular hypertension follow-up management prefer to sign family doctor contract services, and this relationship was varied by SES. To promote the health management of older adults with hypertension through effective family doctor contract services, attentions should be paid to providing regular follow-up services. In addition, family doctors should improve the service quality and design diversified service packages especially for people with high-level education and income.

## Data Availability

The datasets used in the current study are not publicly available due to the confidential policy but are available from the corresponding author on reasonable request.
